# Spatial frequency domain imaging technology based on Fourier single-pixel imaging

**DOI:** 10.1117/1.JBO.27.1.016002

**Published:** 2022-01-24

**Authors:** Hui M. Ren, Guoqing Deng, Peng Zhou, Xu Kang, Yang Zhang, Jingshu Ni, Yuanzhi Zhang, Yikun Wang

**Affiliations:** a Anhui University, Institute of Physical Science and Information Technology, Anhui, China; b Chinese Academy of Sciences, Hefei Institutes of Physical Science, Anhui Institute of Optics and Fine Mechanics, Anhui, China; c The 940th Hospital of Joint Logistic Support Force of Chinese People’s Liberation Army, Department of Sports Medicine, Gansu, China; d Wanjiang Center for Development of Emerging Industrial, Tongling, China

**Keywords:** Fourier single-pixel imaging, spatial frequency domain, optical properties, compressed sensing

## Abstract

**Significance:**

Optical properties (absorption coefficient and scattering coefficient) of tissue are the most critical parameters for disease diagnosis-based optical method. In recent years, researchers proposed spatial frequency domain imaging (SFDI) to quantitatively map tissue optical properties in a broad field of contactless imaging. To solve the limitations in wavebands unsuitable for silicon-based sensor technology, a compressed sensing (CS) algorithm is used to reproduce the original signal by a single-pixel detectors. Currently, the existing single-pixel SFDI method mainly uses a random sampling policy to extract and recover signals in the acquisition stage. However, these methods are memory-hungry and time-consuming, and they cannot generate discernible results under low sampling rate. Explorations on high performance and efficiency single-pixel SFDI are of great significance for clinical application.

**Aim:**

Fourier single-pixel imaging can reconstruct signals with less time and space costs and has fewer reconstruction errors. We focus on an SFDI algorithm based on Fourier single-pixel imaging and propose our Fourier single-pixel image-based spatial frequency domain imaging method (FSI-SFDI).

**Approach:**

First, we use Fourier single-pixel imaging algorithm to collect and compress signals and SFDI algorithm to generate optical parameters. Given the basis that the main energy of general image signals is concentrated in the range of low frequency of Fourier frequency domain, our FSI-SFDI uses a circular-sampling scheme to sample data points in the low-frequency region. Then, we reconstruct the image details from these points by optimization-based inverse-FFT method.

**Results:**

Our algorithm is tested on simulated data. Results show that the root mean square error (RMSE) of optical parameters is lower than 5% when the data reduction is 92%, and it can generate discernible optical parameter image with low sampling rate. We can observe that our FSI-SFDI primarily recovers the optical properties while keeping the RMSE under the upper bound of 4.5% when we use an image with 512×512 resolution as the example for calculation and analysis. Not only that but also our algorithm consumes less space and time for an image with 256×256 resolution, the signal reconstruction takes only 1.65 ms, and requires less RAM memory. Compared to CS-SFDI method, our FSI-SFDI can reduce the required number of measurements through optimizing algorithm.

**Conclusions:**

Moreover, FSI-SFDI is capable of recovering high-quality resolvable images with lower sampling rate, higher-resolution images with less memory and time consumed than previous CS-SFDI method, which is very promising for clinical data collection and medical analysis.

## 1 Introduction

Recent advances in optical imaging show promising applications in biomedical areas due to the potential correlation between the optical properties of biological tissue and biochemical composition.^1^^–^^3^ Among these technologies, spatial frequency domain imaging (SFDI) has warranted closer attention in that SFDI is effective to derive tissue optical parameters in a noncontact manner.^4^^,^^5^ The key of SFDI is acquiring the absorption and scattering coefficients of the tissues by computing on the pattern image that is captured by the hyperspectral imaging cameras on spatially modulated light.^6^

However, the significant issues in SFDI research are as follows: The existing SFDI methods use a camera for data collection, which relies on electronics integration (silicon) and is limited by CCD and CMOS digital technology. In wavebands unsuitable for silicon-based sensor technology, imaging is considerably more complicated, such as the infrared or deep ultraviolet. Cameras with the required resolution at wavelengths where silicon is blind are more expensive. Recently, several attempts have been tried to fix the issue (1) by introducing an SFDI system that is based on compressed sensing theory (CS), named CS-SFDI.^7^ CS method uses a single-pixel detector to collect the images and then reconstructs the images with fewer sampling points. The most frequently used method of CS is to reconstruct the image with optimization-based algorithms.^8^^–^^10^ CS-SFDI replaces the camera with a single-pixel photo detector and collects the measurement matrix of human tissue to reconstruct and demodulate images.

Theoretically, CS-SFDI method passively drops some useful information thus result in high errors in reconstructing images. To improve CS-SFDI method, CS-based parameter recovery algorithm^11^ is proposed to compress the demodulated images before image reconstruction. Unfortunately, in some cases of clinical data with high heterogeneity, CS-based algorithm often produces results that are not distinguishable under low sampling rate.

However, compared with traditional optical imaging technology, single-pixel imaging still has the following two disadvantages: on the one hand, the image quality is far from the level of the current traditional optical imaging system, the image resolution, and the signal-to-noise ratio is low. On the other hand, compression sensing algorithm often requires a lot of computing time and is difficult to reconstruct the details of complex objects. Consequently, most studies in the field of CS-SFDI only focus on collecting small-scaled images.

In this work, we examine the emerging role of single-pixel imaging based on Fourier spectrum in the context of the SFDI image acquisition (FSI-SFDI). First, we replace the binary basis patterns with the grayscale harmonic sinusoid patterns for the single-pixel imaging system, which is proved in our experiments to improve the estimation accuracy of optical coefficients. Second, the proposed method takes full advantage of the sparseness of natural signals in the Fourier domain and recovers higher resolution images with fewer samples. Given the prior knowledge that the main information of an image is concentrated in the low frequency part of the Fourier spectrum,^12^^,^^13^ our method can reconstruct the large-size image by sampling less data point of low-frequency information. To acquire the pattern images, we superimpose Fourier base pattern images on spatial frequency domain images to simulate structured illumination and compressive detection on tissue surfaces: one tissue with sinusoidal light patterns illuminations at two frequencies and three different phases^7^ and another tissue with four-step phase-shifting sinusoid patterns detection.^12^ After obtaining the pattern images of tissue using IFFT method, we use approximate lookup table (LUT) algorithm based on Monte Carlo simulation^14^ to map and demodulate optical properties.

We conduct experiments to evaluate our method. Using the APP-SFDI data set for simulation,^15^^,^^16^ the clearer and more distinguishable images of the optical properties can be generated, and the root mean square error (RMSE) of the optical properties is <5% and SSIM of the optical properties is >0.7 for a 92% reduction in measurements. Beyond that, keeping the same number of samples, high quality and large-size images can be restored with higher time efficiency and the results of our method are more accurate than that of CS-SFDI. It means that FSI-SFDI can use less sampling rate in large-size images to obtain optical properties images with higher definition.

## 2 Approach

### 2.1 Spatial Frequency Domain Imaging

Many researchers utilized SFDI to measure optical coefficients, such as absorption ($\mu_{a}$) and scattering ($\mu_{s}^{\prime }$) properties.^4^ The first step in this process is using illumination of tissue by structured light to generate modulated images (MI).^17^^,^^18^ MI offers an effective way to acquire the spatial modulation transfer function of a turbid medium, which can represent the characteristics of the optical system. After the measurement of optical, we can capture and figure out two spatial frequency images, for example, a DC image (e.g., $0\;{\mathrm{mm}}^{-1}$) and an AC image (e.g., $0.2\;{\mathrm{mm}}^{-1}$), to determine the diffuse reflectance of the sample. Then, SFDI uses diffusion theory or LUT based on Monte Carlo simulations^14^ to extract the optical coefficients from diffuse reflectance images typically.

There are two main types of components used to collect spatial frequency images. One DMD projects 0 and $0.2\;{\mathrm{mm}}^{-1}$ frequency, three-phase spatial structured light to the surface of tissue, and then, one camera collects MIs of the tissue. The AC amplitude $M_{\mathrm{ac}}(x,f_{x})$ and the DC amplitude $M_{\mathrm{dc}}(x,f_{x})$ is solved pixel-by-pixel:

$$M_{\mathrm{ac}}(x,f_{x})=\frac{\sqrt{2}}{3}{\left[{\left(I_{\mathrm{ac}1}-I_{\mathrm{ac}2}\right)}^{2}+{\left(I_{\mathrm{ac}2}-I_{\mathrm{ac}3}\right)}^{2}+{\left(I_{\mathrm{ac}3}-I_{\mathrm{ac}1}\right)}^{2}\right]}^{1/2},$$ (1)

$$M_{\mathrm{dc}}(x,f_{x})=\frac{1}{3}(I_{\mathrm{dc}1}+I_{\mathrm{dc}2}+I_{\mathrm{dc}3}),$$ (2)

where $I_{\mathrm{ac}1}$, $I_{\mathrm{ac}2}$, $I_{\mathrm{ac}3}$ are the backscattered light intensity corresponding to the phases 0π,2/3π,4/3π, with $0.2{\;\mathrm{mm}}^{-1}$ frequency and $I_{\mathrm{dc}1}$, $I_{\mathrm{dc}2}$, $I_{\mathrm{dc}3}$ are the backscattered light intensity corresponding with $0{\;\mathrm{mm}}^{-1}$ frequency, respectively.

The diffuse reflectance of the sample $R_{d}(x,f_{x})$ can be calculated by measuring a standard reflector with existing diffuse reflectance as a reference:

$$R_{d\_\mathrm{ac}}(x,f_{x})=\frac{M_{\mathrm{ac}}(x,f_{x})}{M_{\mathrm{ac},\mathrm{ref}}(x,f_{x})}\cdot R_{d\_\mathrm{ac},\mathrm{ref}}(f_{x}),$$ (3)

$$R_{d\_\mathrm{dc}}(x,f_{x})=\frac{M_{\mathrm{dc}}(x,f_{x})}{M_{\mathrm{dc},\mathrm{ref}}(x,f_{x})}\cdot R_{d\_\mathrm{dc},\mathrm{ref}}(f_{x}),$$ (4)

where $M_{\mathrm{ac},\mathrm{ref}}(x,f_{x})$ and $M_{\mathrm{dc},\mathrm{ref}}(x,f_{x})$ are the AC and DC amplitude of a standard reflector with known diffuse reflectance.

Finally, we employ the precomputed LUT to map the relationship between $R_{d}(x,f_{x})$ and optical properties ($\mu_{s}^{\prime }$ and $\mu_{a}$) by Monte Carlo algorithm.

### 2.2 Fourier-Based Single-Pixel Imaging

Many prior works have been devoted to single-pixel imaging that often captures a scene without a direct line of sight light.^12^^,^^13^ To increase the imaging quality, Fourier-based method for single-pixel imaging is often adopted. Due to the sparsity of the image in the Fourier domain, our proposed method adopts two-dimensional (2D) Fourier single-pixel imaging, using simulated patterns to capture the Fourier spectrum of the scene signal. Therefore, images can be reconstructed through Fourier inverse transform from Fourier spectrum. 2D Fourier transform^19^ and the inverse transform are given as

$$C(f_{x},f_{y})=\int_{-\infty }^{+\infty }\int_{-\infty }^{+\infty }I(x,y)\exp[-j\cdot 2\pi (f_{x}x+f_{y}y)]df_{x}\;df_{y},$$ (5)

$$I(x,y)=\int_{-\infty }^{+\infty }\int_{-\infty }^{+\infty }C(f_{x},f_{y})\exp[j\cdot 2\pi (f_{x}x+f_{y}y)]dx\;dy,$$ (6)

where I(x,y) represents 2D image, $C(f_{x},f_{y})$ is the Fourier spectrum of 2D image, x,y is the Cartesian coordinates in the spatial image domain, $f_{x}$, $f_{y}$ is the Cartesian coordinates in the Fourier domain, and j is the imaginary unit.

Any 2D image is the result of the linear superposition of a series of Fourier base patterns. The weight corresponding to each Fourier base pattern is the Fourier coefficient.^12^ To acquire the Fourier base patterns for 2D inverse Fourier transform (IFT), we use the existing dataset^15^ from APP-SFDI for simulation, superimpose the generated Fourier base pattern on the images, and obtain the final signals of single pixel detector through simulation computation. After the prior study,^20^ the signal detected by a single pixel detector in a simulated environment is the sum of all the pixel intensities. First, to obtain the Fourier coefficients of the object image, a computer is used to generate a Fourier base pattern. The Fourier base pattern is a series of cosine distribution patterns with different spatial frequencies and different initial phases. The Fourier base pattern projected on the tissue surface can be formulated as

$$P(x,y;f_{x},f_{y},Ø)=a+b\cdot \cos(2\pi f_{x}x/M+2\pi f_{y}y/N+Ø),$$ (7)

where a is the average light intensity, b is the contrast, x and y are the rectangular coordinates of the plane in which the target object is located, M and N are the dimensions of the image, $f_{x}$ and $f_{y}$ are the spatial frequencies corresponding to the x and y directions, and Ø is the initial phase.

Therefore, the scattered reflected light $D_{Ø}(f_{x},f_{y})$ that collected from target object O(x,y) can be expressed as follows:

$$D_{Ø}(f_{x},f_{y})=\underset{x,y}{\sum }P(x,y;f_{x},f_{y},Ø)\cdot O(x,y).$$ (8)

To obtain the Fourier spectrum information of a single point in the image, the most commonly used method is the four-step phase-shifting method. Four-step Fourier spectrum acquisition methods are used frequently. 0, 1/2π, π, and 3/2π are assigned to the values of Ø, respectively. Based on the four-step phase-shift algorithm, the Fourier coefficients $C(f_{x},f_{y})$ can be estimated from four values obtained as follows:

$$C(f_{x},f_{y})=(D_{0}(f_{x},f_{y})-D_{\pi }(f_{x},f_{y}))+(D_{\frac{\pi }{2}}(f_{x},f_{y})-D_{\frac{3\pi }{2}}(f_{x},f_{y})).$$ (9)

**Fig. 1:**
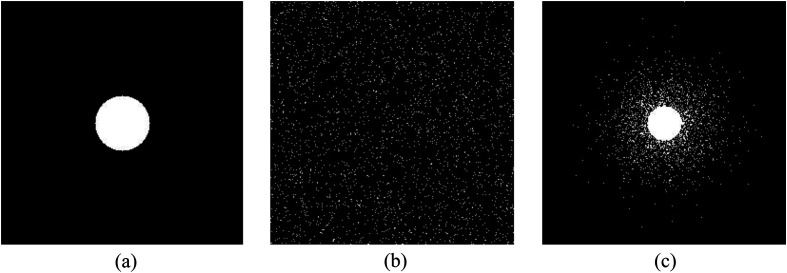
Three sample schemes: (a) circular sampling scheme, (b) random sampling scheme, and (c) variable-density random sampling scheme.

Traditionally, as the four-step phase-shift algorithm is adopted, a complex value in a particular spectrum can be assessed by measuring four projection samples. This means that an M×N pixel image needs to be captured 4×M×N times. Because the Fourier spectrum of the image is conjugate symmetric, an M×N  pixel image only needs to measure 2×M×N projection samples. Previous studies^13^^,^^21^^,^^22^ have confirmed that the main information about objects is concentrated in the lower frequencies of the Fourier spectrum. Therefore, circular acquisition of low-frequencies is the common procedures for reducing sampling rate. However, discarding the spectrum of high frequencies introduces the reduction of the details of the image. To combat this, Wenwen et al. study sparse Fourier single-pixel imaging,^20^ based on variable-density sparse sampling patterns. The probability of sampled distribution is formulated as follows:

$$\rho (r)=\{\begin{array}{cc} 1 & r\leq R\\ {\left(1-r\right)}^{p} & r>R \end{array},$$ (10)

where p is the polynomial coefficient, R is predefined radius of a circular sample in the low frequency, and r represents the Fourier frequency. In the experiment, we measure r by the Euclidean distance from the center point to other points in the image and is normalized to (0,1). Figure 1 shows the forms of three sampling matrices at a sampling rate of 10%.

Sparse Fourier single-pixel imaging can reconstruct high-resolution images using CS optimization algorithms. The optimization algorithms solution of the following equation:

$$\arg\;\min{\left\|Ft-T^{\prime }\right\|}_{2}^{2}+\lambda_{1}{\left\|t\right\|}_{1}+\lambda_{2}\mathrm{TV}(t),$$ (11)

where TV is the total variation operator, $\lambda_{1}$, $\lambda_{1}$ are the loss weight, and t is the object image to be reconstructed. The matrix $T^{\prime }$ is the acquired undersampled spectral data.

### 2.3 Fourier Single-Pixel Imaging-Based SFDI

Previous CS-SFDI methods estimate optical properties in a variety of ways,^7^^,^^11^ which is based on traditional CS algorithm. However, their method generates optical parameters with limited accuracy and fails to produce resolvable images at low sampling rates. To solve this problem, the FSI-SFDI algorithm is proposed in this paper. FSI-SFDI performs SFDI using Fourier single-pixel imaging. To acquire the data, the Fourier base patterns are superimposed on dataset image, which consists of hand images at 0 and $0.2\;{\mathrm{mm}}^{-1}$ frequency, three-phase spatial structured light. The following equation shows the modulated patterns:

$$F(x,y;f_{x},f_{y};f_{k},{Ø}_{1},{Ø}_{2})=\cos(2\pi f_{k}+{Ø}_{1})\cdot P(x,y;f_{x},f_{y},{Ø}_{2}),$$ (12)

where $f_{k}$ is the frequency of SFDI, ${Ø}_{1}$ is the phase of SFDI, and $P(x,y;f_{x},f_{y},{Ø}_{2})$ is the Fourier base pattern. The scattered reflected light $C_{Ø1,Ø2}(f_{x},f_{y},f_{k})$ is collected from target object O(x,y), and can be expressed as follows:

$$D_{Ø1,Ø2}(f_{x},f_{y},f_{k})=\underset{x,y}{\sum }F(x,y;f_{x},f_{y};f_{k},{Ø}_{1},{Ø}_{2})\cdot O(x,y).$$ (13)

**Fig. 2:**
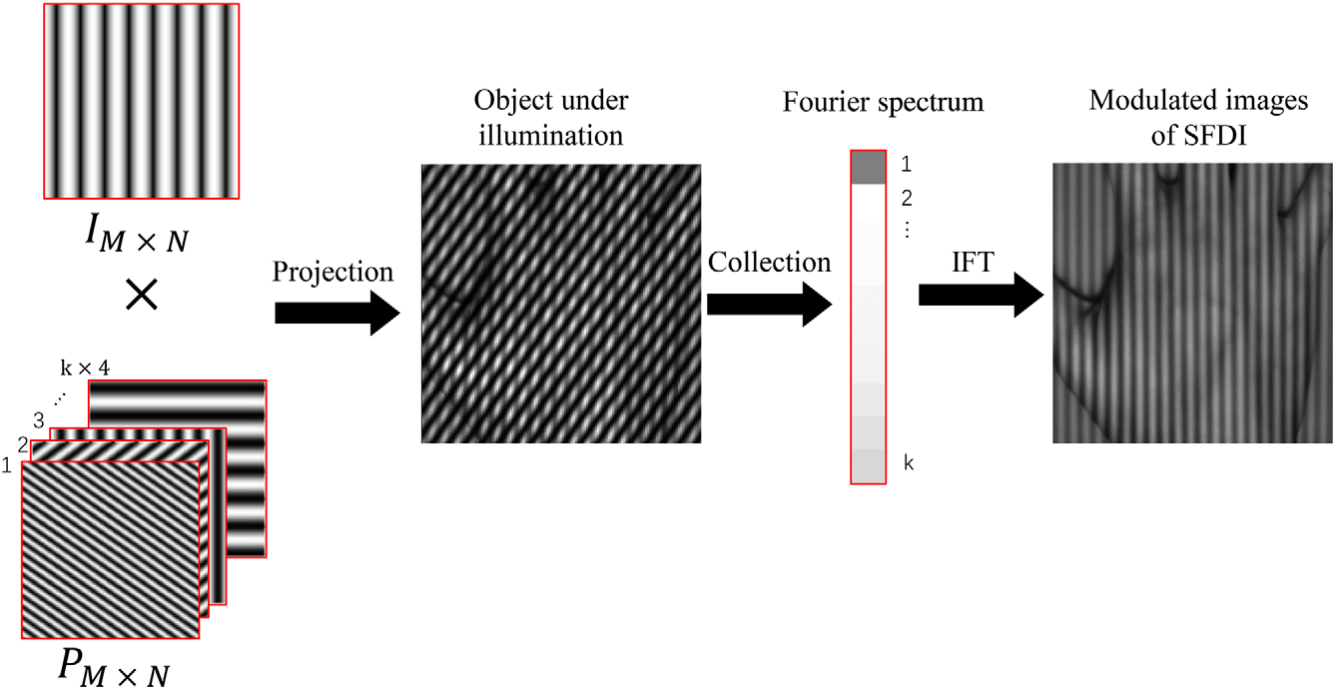
Illustration of image acquisition and reconstruction. The simulated structured pattern images are superimposed on the dataset of SFDI. Single pixel imaging signal is obtained through calculation, which is used to simulate the signal collected by the single pixel detector. After collecting the signal, an MI at a certain spatial frequency and phase can be obtained from the signal by IFT method.

According to Eq. (9), we can compute the Fourier spectrum of the image at a certain spatial frequency and phase. With the Fourier spectrum of the tissues, we utilize IFT to reconstruct the MIs of SFDI. Figure 2 shows an example of image simulation and reconstruction in FSI-SFDI. Because of the large number of patterns required for Fourier single-pixel imaging, it will cost a lot of time and space. To speed up imaging and computing process, a proper solution is to compress the sampling of tissue information. We can take full advantage of the fact that more of the image energy is concentrated in the low frequency of the Fourier spectrum. So we can use circular low frequency sampling, variable density random undersampling, and so on. In the experimental section, we present the efficiency comparison of these sampling methods.

**Fig. 3:**
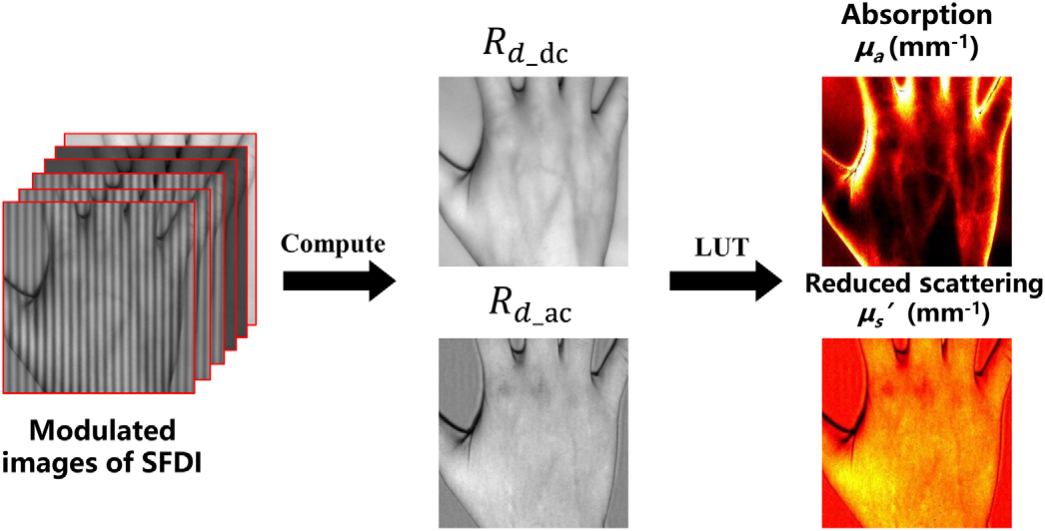
Pipeline of SFDI algorithm. After FSI algorithm which is used to reconstruct multiple modulation images with different phases and frequencies, SFDI can be used to recover optical coefficients.

As shown in Fig. 3, to measure the optical characteristic, we reconstruct the two spatial frequencies and three-phases MIs and calculate AC and DC by Eqs. (1) and (2). Finally, the optical properties: absorption ($\mu_{a}$) and scattering ($\mu_{s}^{\prime }$) properties, can be calculated according to the LUT algorithm or diffusion theory.

### 2.4 Data Simulation

To better verify the effectiveness of the algorithm, we first use the data set to simulate the data on the computer. The data set consists of multiple frequency and phase dorsal patterns. Among the current open resources, APP-SFDI has provided SFDI software and images that are convenient for analysis and testing.^15^ The CS parameter reconstruction algorithm has used this data for experiments and analysis. Therefore, testing on this dataset is reasonable for comparing the performance of the algorithms.

### 2.5 Error Metrics

To verify the error of the results, we choose RMSE for evaluation metrics of our method. Calculation equation of RMSE is obtained as

$$\mathrm{RMSE}=\sqrt{\frac{\sum {\left(\overset{¯}{A}-\overset{¯}{B}\right)}^{2}}{N}}*100,$$ (14)

where $\overset{¯}{A}$ and $\overset{¯}{B}$ are the pixel values of the estimated result and the original image and N is the number of the images.

In addition, to evaluate the accuracy of our results, we adopt structural similarity index to measure the similarity of the two images. Calculation equation of SSIM is

$$\mathrm{SSIM}=\frac{\left(2\times \overset{¯}{A}\times \overset{¯}{B}+C_{1})(2\times \sigma_{AB}+C_{2}\right)}{\left({\overset{¯}{A}}^{2}+{\overset{¯}{B}}^{2}+C_{1})(\sigma_{A}^{2}+\sigma_{B}^{2}+C_{2}\right)},$$ (15)

where $\sigma_{A}$ and $\sigma_{B}$ are the standard deviation of the estimated result and the original image. $\sigma_{AB}$ is the covariance of the estimated result and the original image. $C_{1}$ and $C_{2}$ are the constant terms, and calculation equation of $C_{1}$ and $C_{2}$ is

$$\left\{ \begin{array}{c} C_{1}={\left(K_{1}\times L\right)}^{2}\\ C_{2}={\left(K_{2}\times L\right)}^{2} \end{array},\right.$$ (16)

where $K_{1}$ and $K_{2}$ are the adjustable constant terms. In general, $K_{1}$ is equal to 0.01 and $K_{2}$ is equal to 0.03. L is the data range of the input gray image, where L is 255 for uint8 encoding in our experiments.

## 3 Results

The simulation data use the data set in the APP-SDFI source file, and the source file includes multiple phase and frequency hand modulated illumination images. To compare with the existing methods, this paper uses three phases and two frequencies of dataset as simulations, which are 0π, 1/3π, 2/3π, and 0 and $2\;{\mathrm{mm}}^{-1}$, respectively. And the images are resized to 256×256  pixels.

### 3.1 Comparison of Different Sampling Schemes

**Fig. 4:**
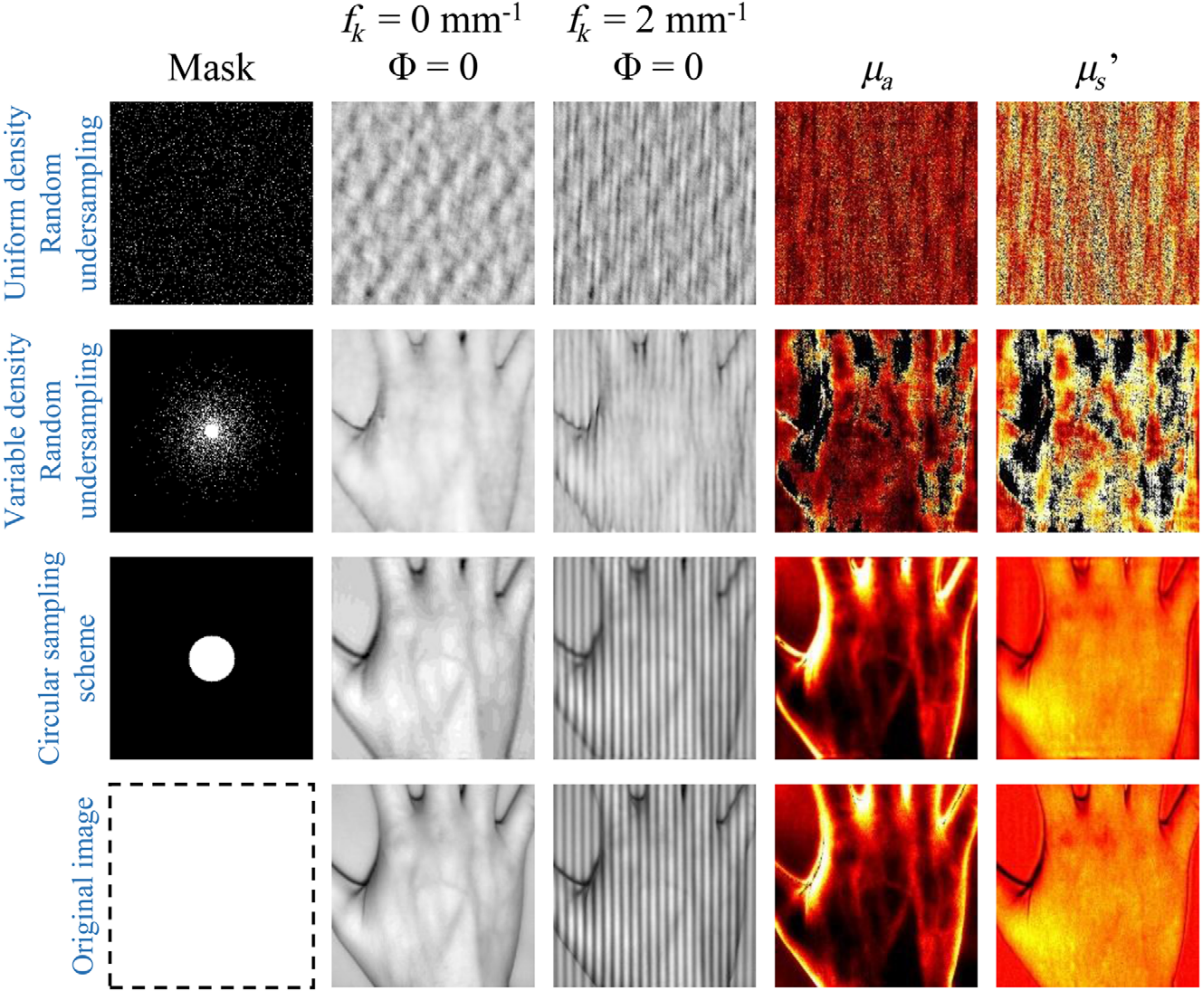
Restoration results of different frequencies and the optical characteristic at a sampling rate of 10%. As presented in this figure, uniform density random undersampling fails to reconstruct resolvable images at low sampling rate. Using variable density random undersampling is better than uniform density random undersampling but cannot significantly improve the accuracy of clinical data with high heterogeneity. On the contrary, it increases the error of the MI when $f_{k}$ is equal to $2\;{\mathrm{mm}}^{-1}$. Circular sampling scheme helps to reconstruct better details with less visible errors than other sampling methods when compared with full sampling.

Since different sampling methods introduce different impacts on the reconstructed MI. To compare the differences between sample schemes, we use different sampling methods at 10% sampling rate to reconstruct the MIs and then generate the optical characteristic map. Three types of sampling schemes and two reconstruction methods have been used to restore the object information. The sampling rate is the ratio of the number of acquisition patterns to the number of image pixels for the convenience of comparison. The results of FSI-SFDI are shown in Fig. 4. Although the sparse Fourier single-pixel imaging has been proved to be effective in recovering the detail information of objects, it is not efficient enough for the situation where the image of skin tissue is flatter. Moreover, the reconstructed images by sparse Fourier single-pixel imaging drop some details of the modulated light, while using circular sampling is more effective to recover better details that are close to the original image.

### 3.2 High Efficient Reconstruction Performance under Different Sampling Rates

**Fig. 5:**
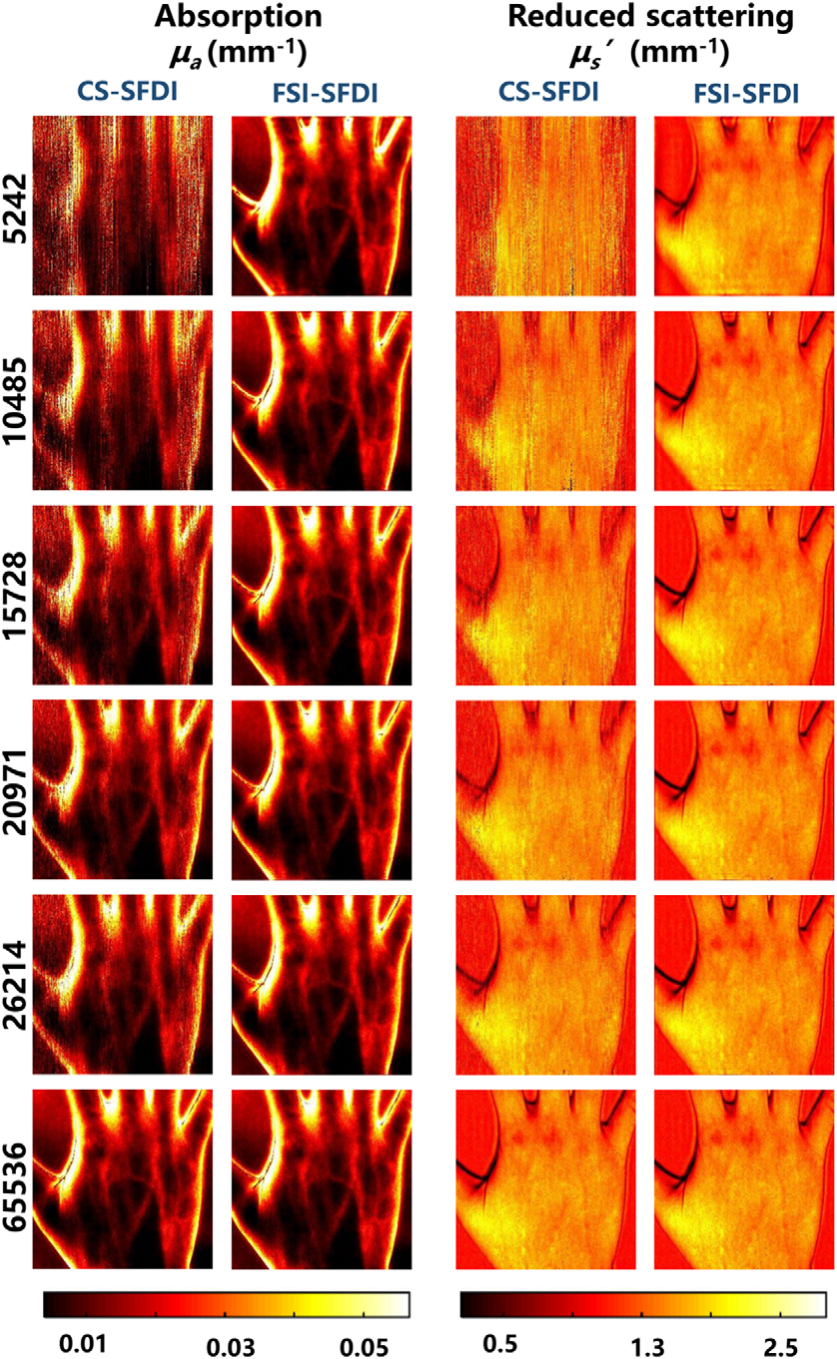
The SFDI based on Fourier single-pixel imaging recovery algorithm image panel by 5242 to 26,214 patterns. Pictures of hand are compressed into 256×256  pixels.

It is very important to obtain more accurate reconstruction results with fewer details losses for exact optical parameter estimation. We have comprehensively compared the performance of our method with that of the CS-SFDI method at different pattern number (i.e., 5242 to 26,214), corresponding to different sampling rates (i.e., 8% to 40%). Figure 5 shows the optical characteristic diagram with pattern number of 5242 to 26,214 by FSI-SFDI and CS-SFDI algorithms. Obviously, at low sampling rate, the image reconstructed by FSI-SFDI method has clear edges and less information loss. At high sampling rate, this method is slightly better than the competitive method. The most interesting aspect of this graph is at 5242 patterns. FSI-SFDI algorithm can clearly preserve the outline of the hand, while the result of CS-SFDI which does not meet the needs of clinical scenarios cannot be distinguished. With lower sampling rate, the FSI-SFDI method is also capable of recovering good details that are clearer than CS-SFDI method.

**Fig. 6:**
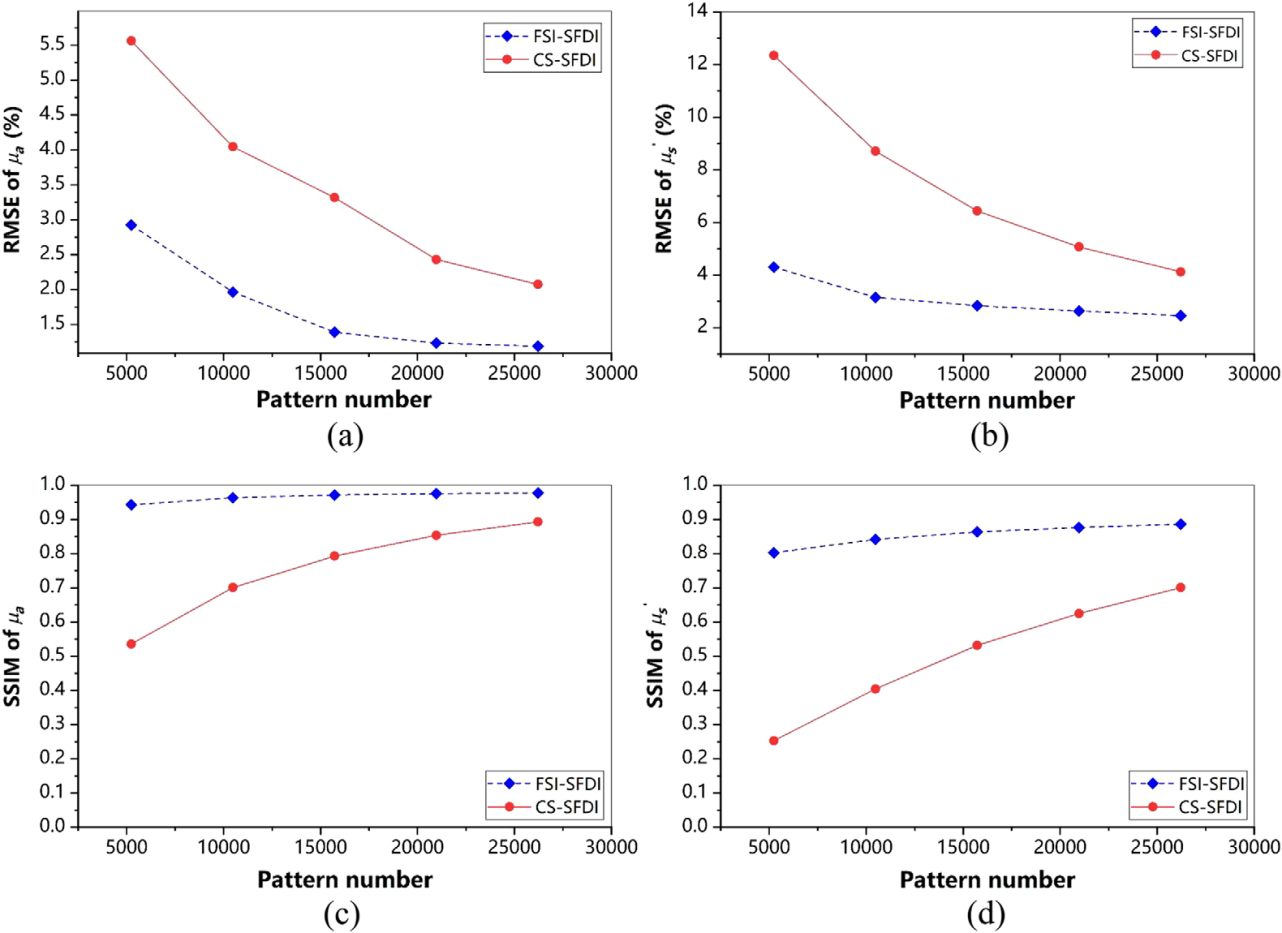
Results comparison of optical properties estimation. (a), (c) The RMSE and SSIM metrics results of $\mu_{a}$ with pattern numbers range from 5242 to 26,214. (b), (d) The RMSE and SSIM metrics results of $\mu_{s}^{\prime }$ with pattern numbers range from 5242 to 26,214. Pictures of hand are resized into 256×256  pixels. Comparison between the original data and reconstructed images for increasing pattern numbers.

According to Fig. 5, the lower sampling rate results in higher reconstruction errors of the MIs, leading to larger estimation error of the optical properties. To evaluate the performance of our method, we measure and compare the RMSE between the estimated optical properties and the original with CS-SFDI method. As shown in Fig. 6, our method achieves much lower errors than CS-SFDI in terms of $\mu_{a}$ and $\mu_{s}^{\prime }$, especially when we use a low sampling rate. The RMSE of FSI-SFDI is <3% ($\mu_{a}$) and <5% ($\mu_{s}^{\prime }$) and the SSIM of FSI-SFDI is >0.9 ($\mu_{a}$) and >0.8 ($\mu_{s}^{\prime }$), respectively.

**Fig. 7:**
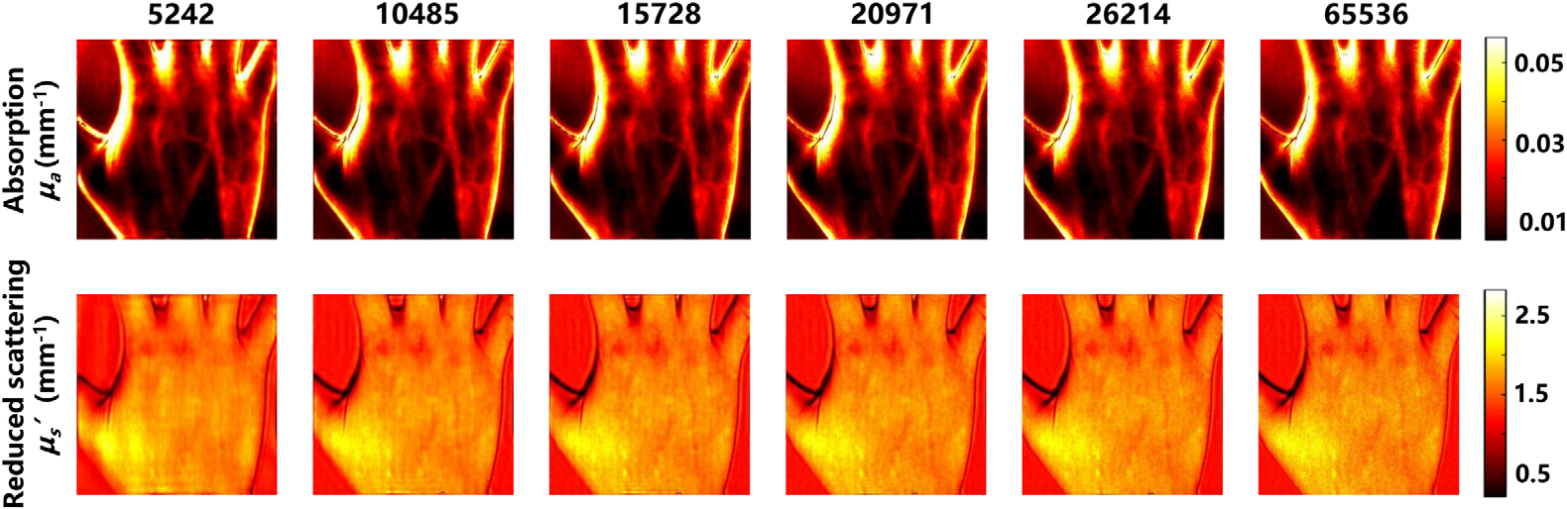
The SFDI based on Fourier single-pixel imaging recovery algorithm image panel with pattern numbers range from 5242 to 26,214. Pictures of hand are compressed into 512×512  pixels.

**Fig. 8:**
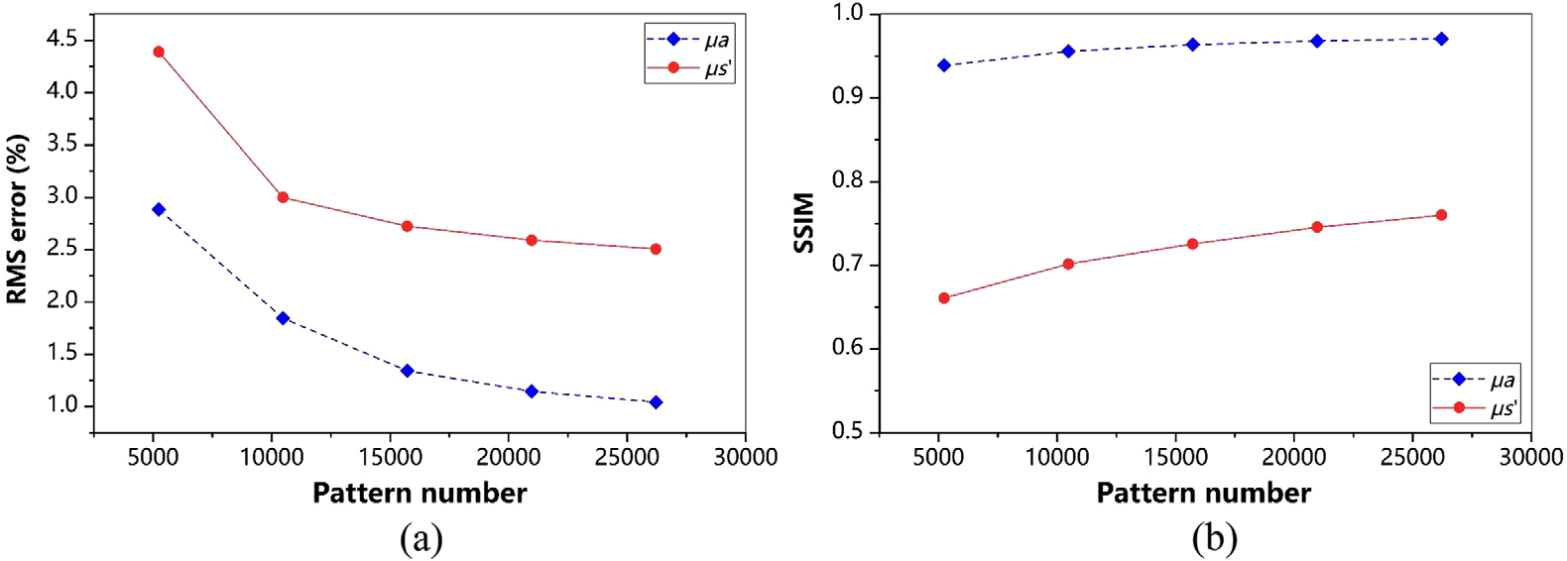
(a) FSI-SFDI RMSE results of data set compressed into 512×512  pixels. (b) FSI-SFDI SSIM results of data set compressed into 512×512  pixels. RMSE for each optical property map obtained using the FSI-SFDI algorithm, compared with the noncompression-based ground truth results.

To evaluate the performance of FSI-SFDI on large-size images, we use an image with 512×512 resolution as the example for calculation and analysis. As the number of modes increases, the computational cost increases. We use the same pattern number 5242 to 26,214 for testing. In qualitative evaluation, as shown in Fig. 7, FSI-SFDI recovers favorable optical characteristic map with few details missed even at 5242 patterns when compared with the results of 100% sampling rate. Quantitatively, we measure the RMSE of the estimated optical properties $\mu_{a}$ and $\mu_{s}^{\prime }$ under different sampling rate. From the curve of Fig. 8, we can observe that our FSI-SFDI primely recovers the optical properties while keeping the RMSE under the upper bound of 4.5%, and the SSIM>0.65.

**Fig. 9:**
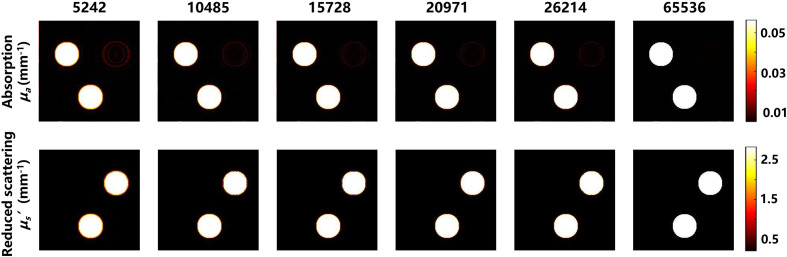
Result of homogeneous two-tone tissue-mimicking phantoms. Comparison between the original data and reconstructed images for increasing pattern numbers.

**Fig. 10:**
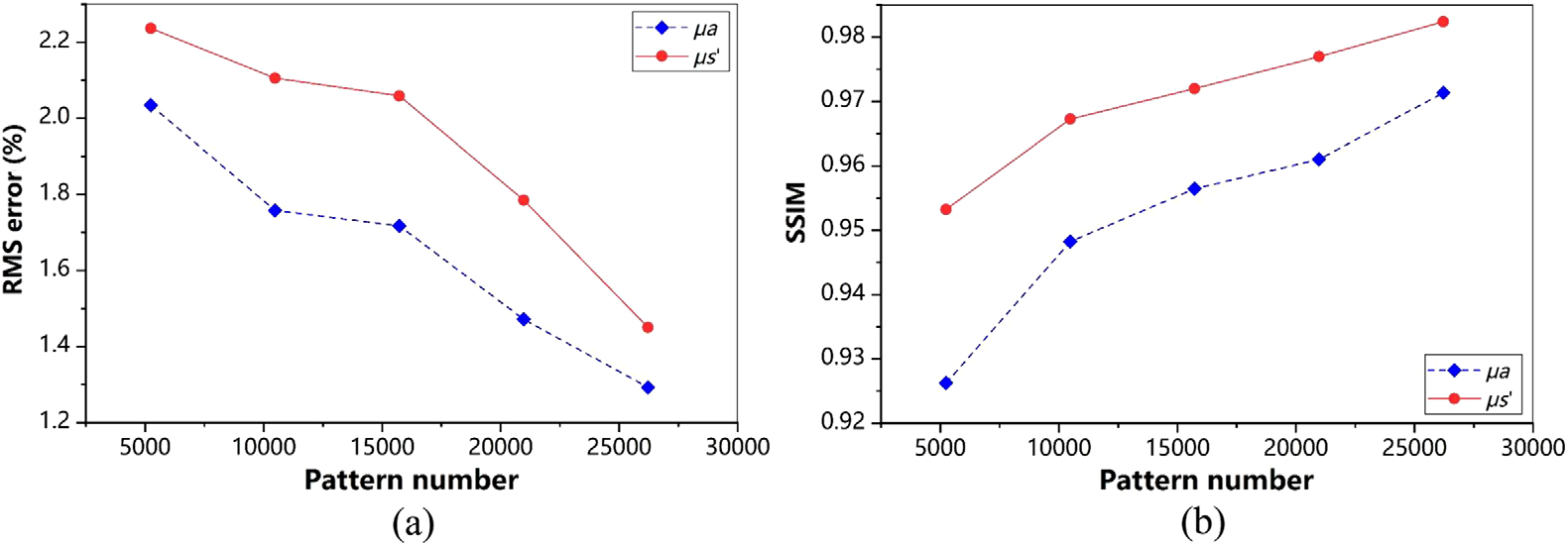
FSI-SFDI RMSE and SSIM results of homogeneous two-tone tissue-mimicking phantoms. (a) RMSE for each optical property map obtained using the FSI-SFDI algorithm, and (b) SSIM for each optical property map obtained using the FSI-SFDI algorithm, compared with the noncompression-based ground truth results.

**Fig. 11:**
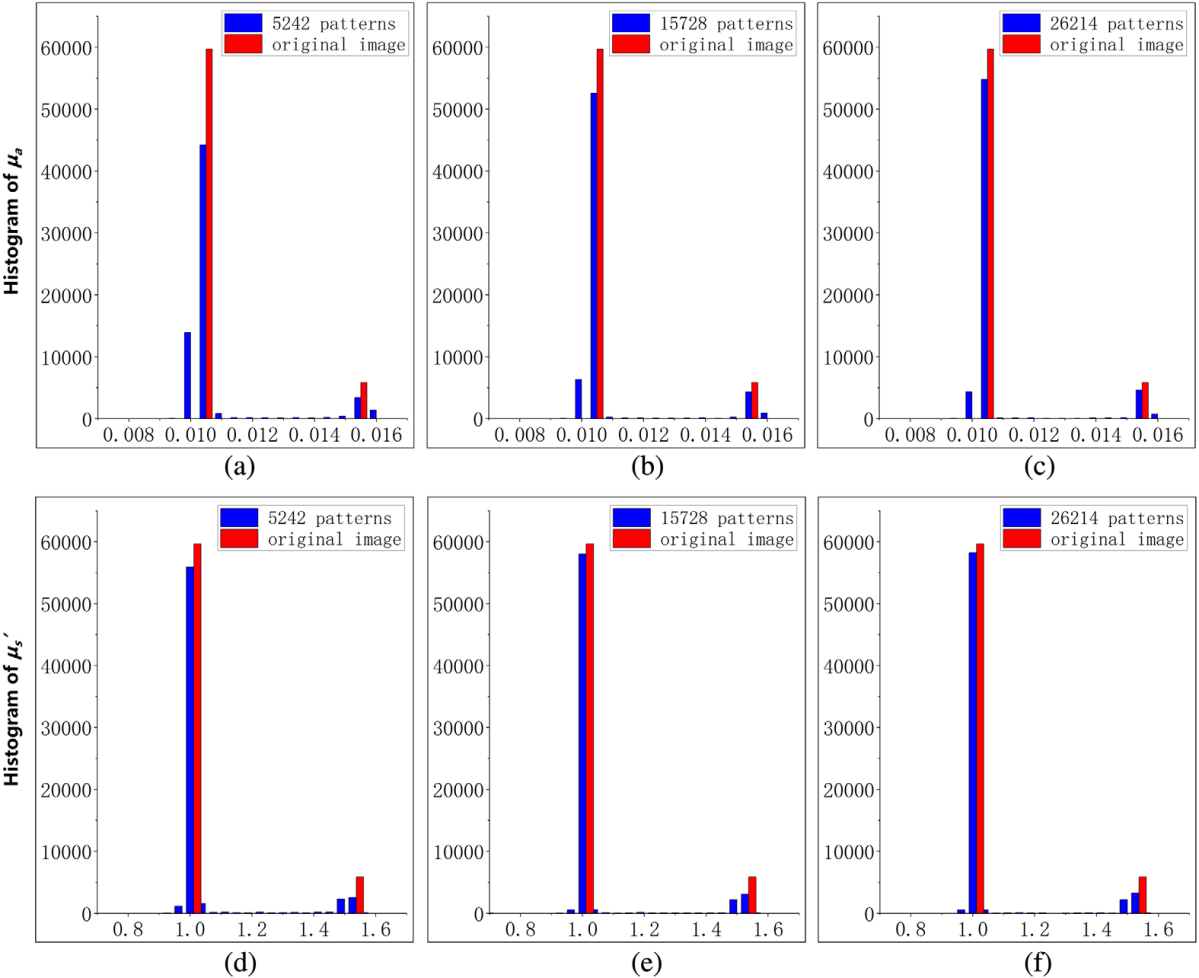
Histogram results of homogeneous two-tone tissue-mimicking phantoms. (a)–(c) Histogram results of $\mu_{a}$ with pattern numbers range from 5242 to 26,214. (c)–(e) Histogram results of $\mu_{s}^{\prime }$ with pattern numbers range from 5242 to 26,214.

To investigate the ability of FSI-SFDI algorithm in estimating optical properties, we utilize homogeneous two-tone tissue-mimicking phantoms. The experiment settings and data of phantom measurement are kept same as Mellors et al.^11^ Specifically, phantom measurements can be simulated using 0 and $0.2\;{\mathrm{mm}}^{-1}$ spatial frequencies images to generate the MI. These simulated data sets have 256×256 resolution, and there are three different optical property anomalies. Figure 9 shows the final anomaly varying and result of the phantom measurements. From the data in Fig. 10, it is apparent that when the data are reduced by 92%, RMSE is lower than 2.5%, and SSIM is greater than 0.9. As can be seen from the histogram in Fig. 11, under the low sampling rate, the results of FSI-SFDI still have relatively high similarity.

### 3.3 Memory and Time Efficiency with Large Resolutions

To compare the efficiency of different reconstruction algorithms, we also evaluate the time efficiency and memory cost of our method when restoring the image signal with 2% sampling rate at 128×128 resolution and 256×256 resolution. The experiment is conducted on a computer server with an Intel Xeon(R) Gold-6124M 2.60 GHz CPU and 128 GB RAM. Table 1 presents the comparison results of memory required and the computation time between two image reconstruction methods. For an image with 256×256 resolution, the CS method takes more than 67,100 ms, while the IFFT method takes only 1.65 ms. For space cost, the CS method requires more than 7000 MB, and IFFT requires 175 MB to reconstruct the image signal.

**Table 1:** Comparison of speed and memory cost for the inversion from Fourier spectrum to images.

Data points	Reconstruction method	Memory (MB)	Time (ms)
128×128	CS	3709.22	17,655
	IFFT	170.43	1.43
256×256	CS	7090.22	67,100
	IFFT	175.59	1.65

**Fig. 12:**
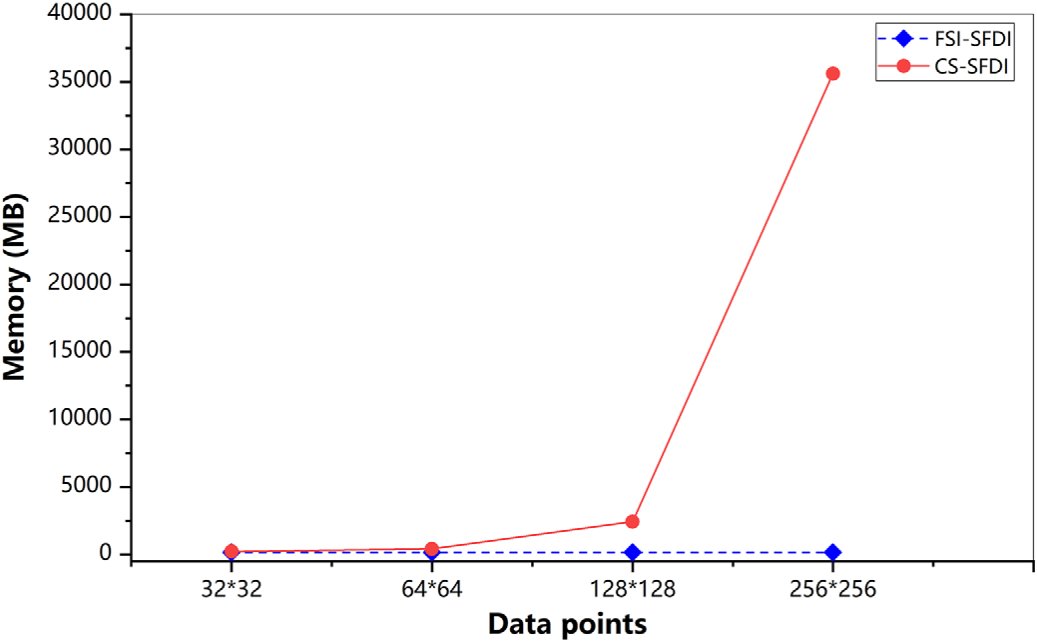
Comparison of memory for CS-SFDI and FSI-SFDI from different data points.

In Fig. 12, we present the memory cost required by the two methods during the computation. Analysis of the figure shows that CS-SFDI method requires the similar amount of computer memory as our FSI-SFDI method when the image resolution is no more than 64×64. When dealing with higher image resolution, CS-SFDI method requires more RAM memory (more than 10 times memory cost of FSI-SFDI) to perform optimization task. In other words, our FSI-SFDI method is technically superior to CS-SFDI in memory cost when applied in cheaper devices, e.g., laptop computer, cell phone, etc.

## 4 Discussion

This work presents a method that uses Fourier single pixel imaging to reconstruct the pattern image based on SFDI theory. We superimpose the two-frequency, three-phase MI onto the image of the spatial frequency domain to simulate the detection of Fourier single pixel imaging on the tissue. Then we reconstruct the optical characteristic map by based method, which effectively improves the accuracy and reduces the time and memory cost compared with previous CS methods.

However, using two-frequencies and three-phase modulated light to acquire six images is still time-consuming. Existing single snapshot algorithms^23^^–^^25^ proposed for reducing the time of acquisition, e.g., one-frequency and one-phase, but downgrade the result accuracies heavily. In the future, it will be possible to consider employing machine learning methods and single snapshot algorithm to reconstruct the optical characteristic map with less patterns.^26^^–^^28^ Moreover, in practical terms, DMD projects grayscale images is slower than binary patterns, further increasing the time cost for collecting information. To alleviate this burden, a programmable DMD operating in binary modes can be used to generate binary (stripe) patterns and then convert the patterns to grayscale (fringe) patterns by employing the defocusing techniques or the spatial low-pass filtering, and the research work of Zhang et al. has suggested the feasibility of path.

**Fig. 13:**
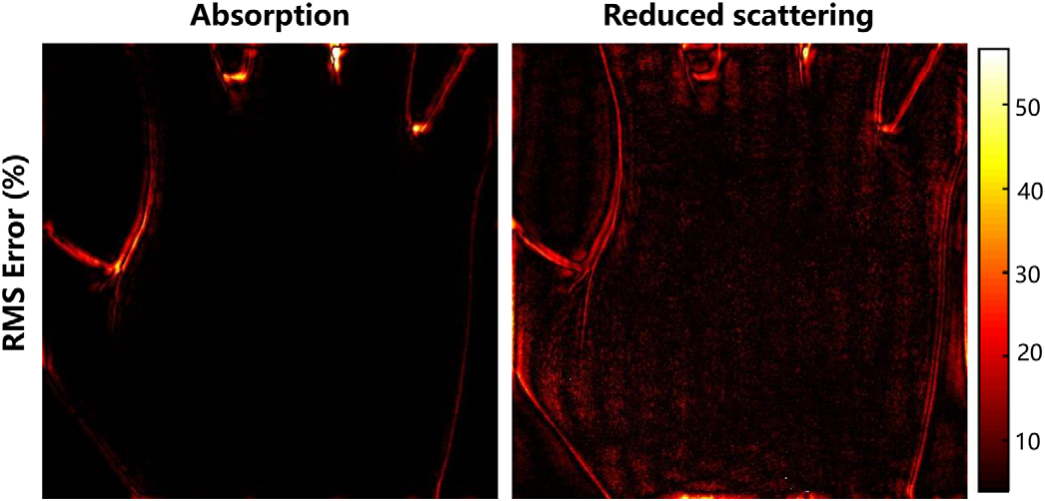
Pixelwise RMSE for 2% sample rate using the FSI parameter recovery algorithm.

Another weakness of our methods is that the high-frequency details are not very clear when the method is dealing with extremely low sampling rate. As Fig. 13 shows, the maximum error is greater than 50% in the area around the edge of the hand due to the loss of high frequency information and the errors in surface curvature. A potential solution is to introduce better and flexible sampling method, which can not only be used to recover the details from low-frequency domain but also high-frequency details. Wenwen et al.^20^ proposed variable density sampling matrix and CS algorithm to achieve super-resolution imaging. Although it has improved the accuracy of the image with more accurate details, the computational efficiency is decreased and it does not apply to MIs. It may in fact demonstrate even greater potency and will be considered in future research.

## 5 Conclusion

In this study, we propose an effective approach for optical parameters estimation via single pixel detector. Our method achieves single-pixel SFDI and achieves the state-of-the art performance. The scope of this contribution is introducing Fourier single pixel imaging method to SFDI (FSI-SFDI), which is more advantageous to generate large size optical characteristic than previous methods. Our system replaces hyperspectral imaging cameras with cheaper single-pixel detector, which is more effective without clear performance reduction. The proposed takes advantage of the sparsity of natural signals in the frequency domain, and collects spectrum data using Fourier single-pixel imaging instead of the CS method, which reduces the computation time and equipment cost for real deployment in a clinical environment.
